# Infant formula feeding practices in a prospective population based study

**DOI:** 10.1186/s12887-016-0754-z

**Published:** 2016-12-08

**Authors:** Hazel Ann Smith, Jonathan O’B Hourihane, Louise C Kenny, Mairead Kiely, Patricia Leahy-Warren, Deirdre M. Murray

**Affiliations:** 1 Paediatrics & Child Health, Clinical Investigations Unit, Cork University Hospital, Wilton, Cork, Ireland; 2 Irish Centre for Fetal and Neonatal Translational Research (INFANT), Cork University Maternity Hospital, 5th Floor, Wilton, Cork, Ireland; 3 Department of Obstetrics and Gynaecology, Cork University Maternity Hospital, 5th Floor, Wilton, Cork, Ireland; 4 School of Food and Nutritional Sciences, Food Science Building, University College Cork, Cork, Ireland; 5 School of Nursing & Midwifery, Brookfield Health Sciences Complex, University College Cork, Cork, Ireland

**Keywords:** * Infant formula, * Infant feeding practices

## Abstract

**Background:**

It is recommended that formula-fed infants are given standard whey-based infant formula throughout the first year of life, unless otherwise advised by healthcare professionals. To our knowledge it has not yet been explored if parents are using a whey-based infant formula throughout the first 12 months of life. Reasons for parental choice of formula are also unknown. Therefore, the objective of this paper was to describe parental administration of whey-based and non whey-based infant formula in the first year of life.

**Methods:**

Data collected as part of the Cork BASELINE Birth Cohort Study examined infant feeding practices at 2, 6 and 12 months of age. Descriptive analysis explored infant feeding practices and parental reasons for changing from a whey-based to a non whey-based infant formula. Multiple logistic regression investigated parental and infant characteristics associated with the use of whey-based infant formula.

**Results:**

In total, 62.4%, 40.4% and 12.8% parent(s) at 2, 6 and 12 months, respectively, gave their infant whey-based infant formula. No parental or infant characteristic was found to consistently influence the use of whey-based infant formula. The most common reason reported by parent(s) for changing their infant’s formula to a non whey-based formula was that they perceived their baby as being hungry.

**Conclusion:**

The majority of parent(s) commence their infants on whey-based formula, but most change to non whey-based formula before 12 months of age. Parental perception of infant satiety and not healthcare advice was the most common reason for changing from a whey-based to a non whey-based infant formula. Additional research is now required to investigate the effect of whey-based and non whey-based infant formula on infant growth.

## Background

Breastfeeding is the internationally recommended method of infant feeding [1] with proven benefits over infant formula feeding [2]. For infants who are not breastfed the Baby Friendly Initiative (BFI), (a programme supported by the World Health Organization and United Nations Children’s Emergency Fund), recommend the use of a standard whey-based infant formula in the first 12 months of life, unless medically indicated by a healthcare professional [3]. This recommendation is supported by Food Safety Authority of Ireland [4, 5]. There are different categories of infant formula and for an infant formula to be described as a standard whey-based infant formula it needs to have a whey:casein protein ratio of 60:40 [6]. Only infant formula labelled as ‘newborn’ or ‘first milk’ meet this definition. Therefore, the majority of infant formula (i.e. soya-based, hydrolysed, follow-on or growing-up infant formula) can be categorised as non whey-based infant formula. To our knowledge it remains unknown what types of infant formula are being used in the first year of life.

The World Health Organization (WHO) recommend that infant feeding practices are regularly monitored [7] but the literature on type of infant formula practices, in comparison to breastfeeding practices, is scant [8–13]. To our knowledge only one study [13] reported the use of standard whey-based infant formula at 6 weeks postpartum but did not examine the use of standard whey-based infant formula throughout the first year of life.

Therefore, in a population based birth cohort, we wished to describe both the use of whey-based and non whey-based infant formula during the first 12 months of life and parental self-reported reasons for infant formula changes. We also examined what parental and infant characteristics were associated with the use of whey-based infant formula.

## Methods

The Cork BASELINE (Babies After SCOPE: Evaluating the Longitudinal Impact using Neurological and Nutritional Endpoints) Birth Cohort (www.baselinestudy.net) is a longitudinal birth cohort study with detailed infant feeding data. Mothers participating in the SCOPE Ireland Study provided their written consent, at 20 weeks’ gestation, for their child to take part in the Cork BASELINE Birth Cohort Study. The SCOPE (SCreening fOr Pregnancy Endpoints) Ireland Study (www.scopestudy.net) ran from 2008 to 2011 and recruited low-risk primigravida mothers with a singleton pregnancy at 15 ± 1 weeks’ gestation. Ethical approval for both the Cork BASELINE Birth Cohort Study (ref ECM5 (9) 01/07/2008) and SCOPE Ireland Study (ref ECM5 (10) 05/02/2008) was provided by the clinical research ethics committee of the Cork Teaching Hospitals [14].

Infants were seen prior to discharge from the maternity hospital after delivery and were followed-up at 2, 6 and 12 months of age. Only term (≥37^+0^ weeks’ gestation) infants who were having at least one bottle of formula a day at the follow-up visits were included for analysis. Mothers that were unable to attend appointments were offered a telephone interview to complete the questionnaires.

Parental body mass index (BMI) was measured at 15 ± 1 weeks’ gestation. Labour and postnatal events, including method of feeding while in the maternity hospital, was collected from the maternity records prospectively by one midwife working on the SCOPE Ireland Study. Method of infant feeding was defined as exclusive breastfeeding (no other fluids), breastfeeding and formula feeding (infant was receiving both breastmilk and infant formula) and formula feeding (infant was exclusively fed formula).

Demographic details including parental nationality and maternal socio-economic status, employment and educational attainment and date of birth, type of maternity care (public or private) and smoking status during pregnancy were collected at 2 months. All maternity care in Ireland is free but women may elect to pay for private maternity care which allows them to select their own consultant, who they will see throughout their pregnancy, during labour and postnatally [15]. Socio-economic status was defined as per the Irish Central Statistics Office guidelines [16].

Information on infant feeding practices was collected at each of the follow-up appointments. Questions included name of current infant formula and, if any other formula had been used previously, and the reasons for changing infant formula. Reasons for changing infant formula was presented as a close-ended question but if the parent’s reason was not available for selection they had the option of giving an open-ended response. Parent(s) were also asked if they had introduced solid food to their infant at each time point and, if applicable, the age (weeks) of the infant when solid food was introduced. All infants, at each time point (2, 6 and 12 months) were categorized as having either a whey-based or non whey-based infant formula. The nutritional data cards provided by the relevant infant formula companies was used to determine if an infant formula was whey-based or non whey-based.

Data were entered prospectively into a secure Internet database. For analysis data was transferred to IBM SPSS Statistics 20 (IBM Corp., Armonk, NY). Categorical data was presented as percentages and continuous variables are shown as mean (SD). The significance level for all analysis was *p* ≤ 0.05.

Descriptive analysis examined infant feeding practices and reasons for using a non whey-based infant formula. Student’s *t*-test investigated continuous variables and chi-square test explored categorical variables for associations with guideline adherence at 2, 6 and 12 months in univariate analysis. Multiple logistic regression examined the significant factors identified in univariate analysis with parent(s) using a whey-based infant formula at 2, 6 and 12 months. Each time point was examined independent of each other.

## Results

**Fig. 1:**
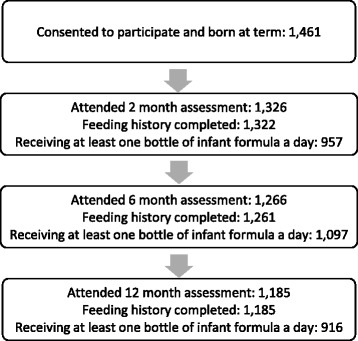
Participant flowchart^*^. In total, from all three follow-up appointments, 1291 infants were included for analysis

From the SCOPE Ireland Study 1,461 mothers, who delivered a term infant, had consented to continue with the BASELINE Birth Cohort Study. Within this cohort 99 parents did not return with their infants for any of the follow-up visits and 71 parents reported that their infant was not given infant formula at least once a day at any of the follow-up appointments. This left, in total, 1291 (88.4%) infants available for analysis (see Fig. 1). We examined for, and found no evidence, of attrition bias between any of the time points. Reasons for parents withdrawing their infant from the study included time and travel constraints.

Nearly all (94.9%) mothers were married or in a stable relationship. The overall mean (SD) birthweight was 3.51 (0.45) kg and the admission rate to the neonatal unit (NNU) was 9.4%, (Table 1).

**Table 1:** Characteristics of study population

Characteristic of study population (*n* = 1,291)	N (%) or mean (SD)
Infant Sex	
Male	653 (50.6%)
Female	638 (49.4%)
Gestational age (weeks) at delivery	40.17 (1.14)
Birth-weight (kg)	3.51 (0.46)
Maternal Age (years)	31.11 (4.36)
Maternal Nationality	
Irish	1078 (83.5%)
Non-Irish	213 (16.5%)
Paternal Nationality^a^	
Irish	1093 (84.7%)
Non-Irish	193 (14.9%)
Mother reported smoking during pregnancy^a^	
No	1132 (87.7%)
Yes	119 (9.2%)
Maternal Tertiary Education	
No	613 (47.5%)
Yes	678 (52.5%)
Maternal Employment Status	
Unemployed	126 (9.8%)
Employed	1165 (90.2%)
Maternity Care	
Public	938 (72.7%)
Private	353 (27.3%)

^a^Percentages do not equal 100 as some mothers did not answer the question

Parental use of whey-based infant formula decreased as the infants got older. At 2 months 62.4% of mothers reported giving their infant a whey-based infant formula. This figure dropped to 40.4% and 12.8% at 6 and 12 months, respectively.

At 2 months the two most popular non whey-based infant formula were those marketed as ‘suitable for hungrier babies’ followed by ‘comfort’ infant formula. This is reflected by the reasons reported by parent(s) for selecting a non whey-based infant formula. The most common reason was that they perceived their infant to be hungry (34.0%) followed by that they didn’t think the current formula suited their infant (17.8%) or the advice of health professionals (12.2%), (Table 2).

**Table 2:** Overall, the most common reported parental reasons for changing infant formula

Reported reason	2 months	6 months	12 months
	*N* (%)	*N* (%)	*N* (%)
Infant was hungry	122 (34.0%)	202 (30.8%)	60 (7.5%)
Advice of healthcare professional	44 (12.2%)	35 (5.3%)	35 (4.4%)
Followed label guidelines	1 (0.3%)	157 (24.0%)	249 (31.2%)

Parental use of ‘follow-on’ infant formula (*n* = 728) and ‘Growing-up milk’ (*n* = 262) were the main reasons for the decreased use of whey-based infant formula at 6 and 12 months. Follow-on infant formula is promoted by infant formula companies as suitable from 6 months of age to complement the period when infants are weaned on to solid food and ‘Growing-up milk’ is promoted as suitable for children from 1 year of age. Both are considered to be non whey-based infant formula.

At 6 months the most common reasons reported by parents for selecting a non whey-based infant formula were parental perception of a lack of infant satiety (30.8%) followed by parent(s) saying that they followed the label advice on the infant formula containers (24.0%), parent(s) reporting that their infant was suffering with reflux (7.0%) and the advice of health professionals (5.3%). At 12 months following label guidelines was the most common (31.2%) reason reported by parent(s) for using a non-whey based infant formula, followed by their perception that their infant was hungry (7.5%) and the advice of a healthcare professional (4.4%), (Table 2).

In investigating, through univariate analysis, which factors are associated with using a whey-based infant formula no maternal, paternal or infant characteristics was consistently associated with using a whey-based infant formula across the three time points; 2, 6 and 12 months. Method of feeding at hospital discharge and maternal tertiary education were associated with using a whey-based infant formula at 2 and 6 months. Maternal nationality was associated with this choice at 2 and 12 months. At 6 months infant sex, maternal age and employment status and maternity care were all significantly associated with infants being fed a whey-based infant formula.

In the multivariable analysis, infants that were formula fed on discharge from the maternity hospital had less odds of having a standard whey-based infant formula at 2 months of age compared to infants that left the maternity hospital exclusively breastfeeding but had since introduced infant formula (aOR = 0.54, 95% CI 0.37–0.79), (Table 3).

**Table 3:** Adjusted odds ratio for using whey-based infant formula at 2 months

*N* = 953	aOR (95% CI)
Method of feeding at hospital discharge	
Exclusive Breastfeeding	Reference
Breastfeeding and formula feeding	0.86 (0.59–1.24)
Formula feeding	0.54 (0.37–0.79)*
Maternal Nationality	
Mother born outside of Ireland	Reference
Mother born in Ireland	0.83 (0.53–1.31)
Paternal Nationality	
Father born outside of Ireland	Reference
Father born in Ireland	0.71 (0.44–1.13)
Maternal tertiary education	
No tertiary education	Reference
Tertiary education	1.28 (0.97–1.68)

*p ≤ 0.05

This trend reversed at 6 months and infants that left the maternity hospital both breastfeeding and formula feeding (aOR = 1.46, 95% CI 1.05–2.03) or were exclusively formula-fed (aOR = 1.52, 95% CI 1.13-2.06) had significantly more odds of receiving a standard whey-based infant formula compared to infants that left the maternity hospital exclusively breastfeeding, (Table 4). We investigated this finding more, to explore why the direction of effect of method of feeding at discharge would differ between the two time points. We found that infants who were exclusively breastfed at discharge from the maternity hospital were more likely to use a whey-based formula at 2 months and then change to a follow-on formula (non whey-based infant formula) at 6 months. Infants that were receiving formula at discharge from the maternity hospital and were given a non whey-based infant formula at 2 months and changed back to a whey-based infant formula at 6 months were changed to a different brand to their first whey-based infant formula.

**Table 4:** Adjusted odds ratio for using whey-based infant formula at 6 months

*N* =1,095	aOR (95% CI)
Infant Sex	
Male	Reference
Female	0.73 (0.57–0.93)*
Maternity Care	
Public	Reference
Private	1.37 (1.04–1.81)*
Method of feeding at hospital discharge	
Exclusive Breastfeeding	Reference
Breastfeeding and formula feeding	1.46 (1.05–2.03)*
Formula feeding	1.52 (1.13–2.06)*
Maternal Age (years)	1.01 (0.98–1.04)
Maternal tertiary education	
No tertiary education	Reference
Tertiary education	1.11 (0.86–1.45)
Maternal employment status	
Unemployed	Reference
Employed	1.57 (0.97–2.54)

*p ≤ 0.05

Maternal smoking during pregnancy was the only characteristic that was significantly associated with an infant formula choice at 12 months. Mothers that smoked had reduced odds of giving their infant a whey-based infant formula compared to non-smoking mothers (aOR = 0.29, 95% CI 0.09–0.93), (Table 5).

**Table 5:** Adjusted odds ratio for using whey-based infant formula at 12 months

*N* =916	aOR (95% CI)
Maternal smoking status during pregnancy	
Non-smoker	Reference
Smoker	0.29 (0.09–0.93)*
Maternal Nationality	
Mother born outside of Ireland	Reference
Mother born in Ireland	1.91 (1.00–3.67)

*p ≤ 0.05

## Discussion

We have shown that the majority of formula-fed infants are not given a whey-based infant formula for the duration of the first year of life. Use of a whey-based infant formula steadily dropped throughout the first year of life, with 40% of formula-fed infants already on a non whey-based infant formula at 2 months of age.

No overall paternal or infant characteristic appeared to influence the use of a whey-based infant formula throughout the first 12 months. How mothers were feeding their infant at discharge from the maternity hospital was the only characteristic associated with the type of infant formula used at both 2 and 6 months. Infants that were exclusively breastfeeding at discharge from the maternity hospital were more likely to be placed on a follow-on formula at 6 months and growing-up milk at 12 months compared to infants that left the maternity hospital either breastfeeding with infant formula or exclusively formula-fed. In comparison infants that left the maternity hospital receiving formula were more likely to be given a non whey-based infant formula that was designed for unsettled babies or babies suffering from colic, reflux etc.

Given the effect of initial breastfeeding, or not, on the use of whey-based infant formula we explored our findings further. Research has shown that there are differences between mothers who breastfed to those that formula-fed [17–19]. Among these factors maternal education has frequently been shown to influence the type of diet children are given [20]. This was also true in our cohort; rates of maternal tertiary education were over four times higher in mothers who initial exclusively breastfed to mothers who did not. Mothers who initially breastfed exclusively but used a follow-on (non whey-based) infant formula at 6 months reported that they were ‘following label guidelines’ and thought that they ‘had to change’ due to their child’s age. These mothers appeared to be seeking for information on infant formula and relied on the advice provided to them by infant formula manufacturers. In comparison, most mothers who formula-fed from birth and switched between whey-based and non whey-based infant formula reported they did so because they felt that the previous infant formula did not suit their infant or their infant did not like the taste of the formula.

Our findings also suggest that parents are not distinguishing between type and brand of infant formula. Some infants who experienced infant formula changes were placed on the same type of infant formula but were given a different brand.

All infants were delivered in the one maternity setting which currently holds a Breast Feeding Hospital Initiative (BFHI) certificate of commitment. This certificate is awarded to settings who currently do not hold BFHI status but have declared their intention to work towards achieving BFHI accreditation. The BFHI requires that mothers who chose to give their child any infant formula are taught, individually, about formula preparation, handling, storage and feeding but does not include educating parents on current recommendations on type of infant formula [21]. As UNICEF supports the use of a whey-based infant formula when breastmilk is not available [3] this study would suggest that an evaluation of current standards for parental education should encompass all aspects of infant formula feeding, including what type of infant formula to use.

The few studies that have examined infant formula feeding practices have mostly focussed on frequency of formula changes rather than formula constituents. Early studies, carried out in 1980 [11] and 1995–1996 [10], explored changing from a ‘standard’ to a ‘special’ formula but as these studies did not define their groups it is difficult to evaluate their findings. An Israeli study undertaken in four maternal and child health care centres between 2002 and 2003 found that 47% of infants experienced a formula change in the first 6 months of life. Most of the formula changes were to another cow’s milk based formula (not defined) and, on average, the first change occurred at 3 months [8]. The EDEN (Étude des Déterminants pré et postnatals du développement et de la santé de l’Enfant) mother-child cohort reported on the effect of the predominant choice of infant formula in the first 4 months of life on infant growth. The study found that 26% of infants had experienced two or more formula changes in the first 4 months of life. No significant relationship was found between growth and predominant formula (predominant infant formula was a mixture of whey-based and non whey-based infant formula) [9].

One Irish study did descriptively report the type of infant formula used by parent(s) 6 weeks following delivery [13]. The study involved term (≥37^+0^ weeks’ gestation) singleton infants born with a birthweight of 2.5 kg or greater. Out of the total sample of 450 infants, 368 (81.8%) infants were formula-fed at 6 weeks of age and just over half (*n* = 197; 53.6%) were being given a standard whey-based infant formula. Nearly half (*n* = 181, 49.2%) of all infants had experienced at least one formula change. For infants whose formula was changed, either to a whey-based or non whey-based infant formula, parental reports of their infant’s increased hunger/feeding frequency of 2–3 h was the most (54.8%) reported reason. The study did not provide any information on the initial type of infant formula, or feeding history on type of infant formula after 6 weeks of age.

An analysis of data from the Infant Feeding Practices Study II (IFPS II) examined the effect of marketing, direct or through health professionals, on formula changes [12]. The authors reported that formula changes were made for mainly non-health reasons (health reason was defined based on stool characteristic or diarrhoea, vomiting and fussiness). In our study, parental perception of their infant’s appetite was the most reported reason for changing infant formula. This was followed by advice the mother had received from a healthcare professional (nurse or doctor) at either a routine appointment (such as vaccination or developmental assessment) or if the mother specifically requested to see a doctor over a concern with her child. The influence of healthcare professionals on formula feeding practices was mainly observed at 2 months of age. At 6 and 12 months more mothers reported that they followed the label guidelines on the infant formula containers or from the helpline of the infant formula company than advice from a healthcare professional in their reason for changing their infant’s formula. This brings to attention the influence of marketing from infant formula companies on changing infant formula.

There are limitations to this study as we did not collect information on parental or healthcare professional knowledge on infant formula feeding guidelines. It therefore remains unknown if parents and healthcare professionals are aware of international guidelines on type of infant formula. We have, however, reported which formula, based on BFI guidelines, infants are exposed to in the first year of life. The WHO recommend that all infant feeding (breast- or formula-feeding) is monitored and this paper addresses the current gap in our knowledge on formula-feeding practices. Our results show that parental reports of infant satiety and marketing from infant formula companies but not advice from healthcare professionals influenced their decision on what type of infant formula to purchase. This research now needs to be followed-up by examining infant health outcomes of infants who received whey-based or non whey-based infant formula.

## Conclusion

We found that most formula-fed infants are given a non whey-based infant formula in the first year of life. The effect of this feeding practice on infant health is unknown. Further research needs to be undertaken to evaluate the appropriateness and value of current guidelines on type of infant formula. It also needs to be investigated what knowledge health care professionals and parent(s) have on the current guidelines on type of infant formula.

## Abbreviations

aOR: Adjusted odds ratio
BFHI: Breast feeding hospital initiative
CI: Confidence interval
WHO: World health organization
